# Rethinking the tools in the toolbox

**DOI:** 10.1186/s12984-022-01041-3

**Published:** 2022-06-20

**Authors:** T. George Hornby

**Affiliations:** 1 Department of Physical Medicine and Rehabilitation, Indiana University School of Medicine, 4141 Shore Drive, Indianapolis, IN 46254 USA; 2 Rehabilitation Hospital of Indiana, Indianapolis, IN USA; 3 Departments of Physical Medicine and Rehabilitation, Northwestern University Feinberg School of Medicine, Chicago, IL USA

**Keywords:** Robotic-assisted gait training, Locomotion, Rehabilitation

## Abstract

The commentary by Dr. Labruyere on the article by Kuo et al. (J Neuroeng Rehabil. 2021; 18:174) posits that randomized trials evaluating the comparative efficacy of robotic devices for patients with neurological injury may not be needed. The primary argument is that researchers and clinicians do not know how to optimize training parameters to maximize the benefits of this therapy, and studies vary in how they deliver robotic-assisted training. While I concur with the suggestion that additional trials using robotic devices as therapeutic tools are not warranted, an alternative hypothesis is that future studies will yield similar equivocal results regardless of the training parameters used. Attempts are made to detail arguments supporting this premise, including the notion that the original rationale for providing robotic-assisted walking training, particularly with exoskeletal devices, was flawed and that the design of some of the more commonly used devices places inherent limitations on the ability to maximize neuromuscular demands during training. While these devices arrived nearly 20 years ago amid substantial enthusiasm, we have since learned valuable lessons from robotic-assisted and other rehabilitation studies on some of the critical parameters that influence neuromuscular and cardiovascular activity during locomotor training, and different strategies are now needed to optimize rehabilitation outcomes.

The commentary by Dr. Labruyere focuses on the question of how robotic rehabilitation devices should be utilized or studied, and was instigated in part by the work of Kuo et al. [1] using the Lokomat, an exoskeletal robotic device introduced earlier this century. In citing the recent Cochrane review by Mehrholz et al. [2] on locomotor training using electrotechnical devices in individuals with stroke, Dr. Labruyere questions the conclusions that further trials are warranted, particularly for robotic-assisted training paradigms. Rather, using the Kuo article as an example, he believes that robotic training parameters such as body weight support, treadmill speed, and the amount of robotic assistance vary substantially across and within different studies or between therapists, and these differences may influence the efficacy of the training delivered. The primary argument is that manipulating these settings individually or in combination varies the neuromuscular demands of walking training, and therapists and researchers should work towards optimizing these training parameters to maximize patient outcomes. In referencing a concluding remark from the Cochrane review, Dr. Labruyere suggests that additional, larger randomized controlled trials using robotic devices are likely not needed, perhaps until we determine the best training conditions for specific patients.

Dr. Labruyere and I certainly share the opinion that, 20 years ago, robotic devices were introduced amid substantial enthusiasm that these new tools could provide greater amounts of walking practice for individuals with neurological injury, while simultaneously decreasing the physical burden on therapists. We also agree that, after two decades of evaluation of their potential efficacy, further randomized trials are not needed. However, we have very different opinions on why such studies are unwarranted.

My concern is that the results of further randomized trials using robotic-assisted training strategies will result in similar equivocal results that we have witnessed over the past 20 years. Large randomized trials are often initiated on the promise of findings from smaller trials that demonstrate a relative benefit (i.e., greater effect size) of an experimental intervention as compared to an alternative strategy. However, in published randomized trials using robotic devices, the results are consistently inconsistent and inconclusive. There are certainly some positive studies hinting towards the comparative efficacy of robotic-assisted training [3, 4], although those studies often utilize a comparison intervention that provides limited walking training (i.e., conventional therapy). That message is important; more walking practice often results in better walking outcomes [5, 6]. In many studies, however, there are no differences in primary or secondary walking outcomes. Rather, some authors often highlight small, non-significant differences that slightly favor robotic-assisted therapy [7, 8], emphasize various secondary outcomes that might be significant (gait symmetry or body composition) [9, 10], or use non-standard statistical procedures to achieve significant differences [11, 12]. That “spin” is often interpreted by clinicians and researchers as an indication of the superiority of robotic-assisted training, despite the collective evidence demonstrating limited comparative efficacy of such training (see [13, 14] for review). Conversely, in some of the early, larger randomized trials comparing robotic-assisted therapy to alternative interventions, particularly those that focus on task-specific walking with therapist assistance, the results demonstrate statistically significant differences favoring the alternative strategy; that is, robotic-assisted training is worse [15–17]. More recent studies still suggest no additional benefits of robotic-assisted training on mobility outcomes, even using more sophisticated training algorithms (i.e., reduced guidance or path control) [18–21]. There is very little indication that additional, larger randomized trials would yield different results. Indeed, Dr. Labruyere and I reach the same conclusion, although our rationales are very different.

Where Dr. Labruyere and I disagree is whether the training parameters that can be manipulated during robotic-assisted training need to be further evaluated to optimize the efficacy of this therapy. Dr. Labruyere cites ample data to suggest that lower limb electromyographic (EMG) activity is modulated by varying training conditions in the device used by Kuo and colleagues (i.e., Lokomat). Manipulation of specific parameters certainly can increase the neuromuscular demands of walking training, which should be the goal of therapy sessions. However, three of the important predictors in the Kuo study, including body-weight support, speed, and number of days to attend 12 sessions, are unrelated to the exoskeletal device. Importantly, the robotic exoskeletal device primarily provides compliant assistance for limb swing, with some stance-phase assistance [22]. While critical for successful ambulation, limb swing represents a relatively minor fraction of the energetic demands of upright ambulation (10%) [23]. Rather, propulsion, stance control, and postural stability encompass a much larger percentage of the metabolic costs of walking [24–28]. Unfortunately, the Lokomat, which is a stationary exoskeletal device, does not provide compliant assistance for these biomechanical demands, or in selected cases restricts them such that volitional effort may be reduced. For example, manipulating body weight support modulates the neuromuscular demands of extensor muscles for stance control [25, 29], with minor contributions from the exoskeleton, although such support is manipulated separately from the robotic device. Gait speed typically requires alterations in propulsive forces during normal treadmill walking [30, 31] and also can be manipulated separately, although the anterior-posterior pelvic restraint of the exoskeleton limits the need to generate those forces. The neuromuscular demands required to maintain lateral postural stability are also minimized in the Lokomat with the lateral restraints. The combined restrictions in movements can substantially reduce the metabolic costs observed during standing in the robotic device as compared to standing without the exoskeleton [22]. During walking, previous data suggest that metabolic costs are reduced even more dramatically in the Lokomat as compared to treadmill walking without physical assistance or therapist assistance only as needed [6, 22, 32]. Reducing the guidance force for limb swing using more advanced training algorithms does not substantially alter the metabolic costs of walking [33], particularly as compared to unassisted conditions. Unfortunately, these findings are consistent during robotic-assisted stepping using an elliptical device [32]. Given the important role of increasing energetic demands during walking training, specifically with attempts to achieve higher cardiovascular intensities [13, 34, 35], reduced metabolic costs during robotic-assisted walking is likely a major factor limiting the efficacy of this strategy, and fine-tuning the robotic training parameters will likely not influence outcomes.

Dr. Labruyere’s overarching desire to optimize training parameters is important though, as he suggest researchers should direct efforts towards identifying the variables that will increase volitional neuromuscular demands. The primary difference in our opinions is how to achieve those higher neuromuscular, and subsequently cardiovascular, demands. One strategy would be to encourage participants to work as hard as they can during robotic-assisted training, which was previously shown to result in equivalent metabolic demands early during a 10-min walking bout as compared to walking with therapist assistance-as-needed [22]. In that study, however, abnormal muscle patterns were observed during the swing-phase of the gait cycle, and the metabolic costs gradually reduced over the 10-min bout [6, 22]; these data support the hypotheses of “slacking” [36, 37] or the “principle of laziness” [38] in which individuals reduce volitional effort when it is not required. Alternatively, therapists could remove patients from the robotic device but continue to focus on walking, particularly at higher cardiovascular demands. This latter strategy seems to improve walking outcomes fairly consistently across ambulatory or non-ambulatory individuals with neurological injury [13, 34, 35].

Given these limitations, the value of robotic-assisted training in the rehabilitation of patients with neurological injury is questionable, particularly when compared to walking training with therapist assistance only as needed. When attempting to mobilize patients with substantial disability, the number of therapists could certainly be reduced with robotic assistance. However, given the exorbitant purchase and maintenance fees of these devices, their value is unclear, particularly when less expensive, elastic devices could also reduce therapist’s exertion [23–25] A common counterargument is that robotic devices control kinematic trajectories, which was previously thought to be important for retraining ambulation [39]. However, data from therapist-assisted training studies focused on normalizing kinematics [40, 41], or exoskeletal-assisted training studies [42, 43], suggest gains in gait function and kinematics were not superior to, or worse than, walking training strategies that do not focus on kinematics. Indeed, recent randomized trials [44, 45] and implementation efforts [35, 46, 47] in severely impaired patients post-stroke indicate that attempts to maximize the amount of stepping practice at higher cardiovascular intensities without focusing on kinematics results in significant gains in mobility outcomes and gait kinematics. If available, robotic-assisted devices may be helpful very early in the recovery process for individuals with substantial disability, although efforts should be made to remove the patients from the device as rapidly as possible to maximize volitional engagement [48].

Additional studies that evaluate techniques to enhance locomotor function for individuals with neurological injury are certainly needed to advance our field, but these studies should not use the same robotic tools that have not shown a clear benefit after 20 years of evaluation. We certainly learned valuable lessons from robotic-assisted training studies of what can improve walking, such as providing large amounts of task-specific (i.e., stepping) practice. We also learned valuable lessons from these tools of what not to do, including providing assistance to normalize kinematics and keeping cardiovascular intensities low. While most studies have focused on stationary robotic devices, the available data supporting the utility of mobile robotic devices are similarly inconsistent.^7,12,49−53^. As such, a new generation of robotic devices are needed for our field to move forward. Such devices need to be far more agile, including the ability to seamlessly don or doff these devices to allow independent ambulation and the capacity to navigate real-world environmental barriers while maintaining postural stability. Such devices would transform the landscape of mobility options used in the home and community setting, rather than utilized primarily as therapeutic tools. Unfortunately, such devices are not currently available and after two decades of evaluation of robotic-assisted training strategies, it’s time to rethink the tools in our toolbox. We need different tools.

## Data Availability

Not applicable.
